# Tree Plantation Systems Influence Nitrogen Retention and the Abundance of Nitrogen Functional Genes in the Solomon Islands

**DOI:** 10.3389/fmicb.2015.01439

**Published:** 2015-12-22

**Authors:** Frédérique Reverchon, Shahla H. Bai, Xian Liu, Timothy J. Blumfield

**Affiliations:** ^1^Instituto de Ecología A.C., Red de Estudios Moleculares AvanzadosXalapa, México; ^2^Environmental Futures Research Institute, School of Natural Sciences, Griffith University, NathanQLD, Australia; ^3^Faculty of Science, Health, Education and Engineering, University of the Sunshine Coast, MaroochydoreQLD, Australia; ^4^Environmental Futures Research Institute, School of Environment, Griffith University, NathanQLD, Australia; ^5^Forestry College, Fujian Agriculture and Forestry UniversityFuzhou, China

**Keywords:** *Tectona grandis*, *Flueggea flexuosa*, mixed species plantations, δ^15^N, tropical soil, qPCR

## Abstract

Tree mono-plantations are susceptible to soil nutrient impoverishment and mixed species plantations have been proposed as a way of maintaining soil fertility while enhancing biodiversity. In the Solomon Islands, mixed species plantations where teak (*Tectona grandis*) is inter-planted with a local tree species (*Flueggea flexuosa*) have been used as an alternative to teak mono-plantations and are expected to increase soil microbial diversity and modify microbial biogeochemical processes. In this study, we quantified the abundance of microbial functional genes involved in the nitrogen (N) cycle from soil samples collected in teak, flueggea, and mixed species plantations. Furthermore, we measured soil properties such as pH, total carbon (C) and total N, stable N isotope composition (δ^15^N), and inorganic N pools. Soil pH and δ^15^N were higher under teak than under flueggea, which indicates that intercropping teak with flueggea may decrease bacterial activities and potential N losses. Higher C:N ratios were found under mixed species plantations than those under teak, suggesting an enhancement of N immobilization that would help preventing fast N losses. However, inorganic N pools remained unaffected by plant cover. Inter-planting teak with flueggea in mixed species plantations generally increased the relative abundance of denitrification genes and promoted the enrichment of *nosZ*-harboring denitrifiers. However, it reduced the abundance of bacterial *amoA* (ammonia monooxygenase) genes compared to teak mono-plantations. The abundance of most denitrification genes correlated with soil total N and C:N ratio, while bacterial and archeal nitrification genes correlated positively with soil NH_4_^+^ concentrations. Altogether, these results show that the abundance of bacterial N-cycling functional guilds vary under teak and under mixed species plantations, and that inter-planting teak with flueggea may potentially alleviate N losses associated with nitrification and denitrification and favor N retention. Mixed plantations could also allow an increase in soil C and N stocks without losing the source of income that teak trees represent for local communities.

## Introduction

In tropical countries such as the Solomon Islands where deforestation rates are high, tree plantations are seen as a way to counteract soil degradation by restoring vegetation cover while decreasing the existing pressure on native forests (Wolfe et al., 2015). However, most established tree plantations are mono-plantations that can cause the same environmental problems as other monoculture systems, namely higher pest or disease occurrence and a modification of biogeochemical cycles and nutrient availability (Rachid et al., 2013). In order to maintain soil fertility and to enhance biodiversity, mixed plantations are being promoted and are expected to reduce plant competition for nutrients and increase soil carbon (C) and nitrogen (N) pools (Montagnini, 2000; Balieiro et al., 2008; Vigulu, 2015). Mixed-species systems are also expected to enhance soil microbial diversity by increasing the variety of carbon (C) based resources and the heterogeneity of spatial patterns in soil properties (Thevathasan and Gordon, 2004).

Many essential soil processes are primarily mediated by microbial communities (Bissett et al., 2013). Land management practices and changes in plant species composition are known to impact microbially driven processes in soils through the alteration of bacterial communities that will thereby modify nutrient availability to plants or nutrient losses from the ecosystem (Chen et al., 2003; Kourtev et al., 2003; Lindsay et al., 2010). Soil microorganisms play a central role in organic matter decomposition and in the cycling of major plant nutrients, including N (Hallin et al., 2009; Bissett et al., 2011). Yet, the importance of soil microbial diversity is often overlooked when establishing forest plantations (Levy-Booth and Winder, 2010). With the growing concerns about intensive mono-plantations comes an increasing interest in the management of soil fertility and soil bacterial communities to enhance tree growth and productivity (Lacombe et al., 2009). However, how the establishment of mixed plantations influences the functions of soil bacterial communities, as measured through microbial functional genes (MFGs), and how this relates to nutrient cycling remains to be understood.

In the Solomon Islands, teak (*Tectona grandis* L.f.) is often grown in smallholder plantations in order to rehabilitate the logged-over rainforests while providing a source of income to landowners (Vigulu, 2015). Teak is an economically important timber tree species grown in tropical and sub-tropical countries for its highly durable hardwood (Miranda et al., 2011). Mostly cultivated in monoculture plantations in 20–40 years rotation, its height can reach more than 20 m at maturity (Ladrach, 2009). Teak grows well on a broad range of soils but its growth has been reported to be optimal on deep and well-drained sandstones, with neutral or acid pH and high calcium, phosphorus, potassium, N, and organic matter contents (Kadambi, 1972). Currently, a new plantation system is being introduced in the Solomon Islands where teak is intercropped with a local tree species (*Flueggea flexuosa* Muell. Arg.) in order to overcome the reluctance of growers to thin pure teak stands. Flueggea, a small to medium tree typically 10–16 m tall, is traditionally used for house building and fencing in the Solomon Islands (Thomson, 2006). Flueggea was considered as a good candidate species for intercropping with teak as roots from both trees seem to occupy different soil depth. While teak has extensive horizontal and vertical roots and occupy a large portion of the soil volume, flueggea’s root system usually develops laterally, near the soil surface (Thomson, 2006; Vigulu, 2015).

The implementation of mixed-species systems is likely to influence nutrient cycling and the abundance of MFG associated with nutrient cycling (Rachid et al., 2013). Therefore, we aimed to determine the abundance of MFG involved in N cycling under teak mono-plantations, flueggea mono-plantations and mixed-species systems and evaluate differences in soil N pools due to tree cover. To accomplish this, we assessed the abundances of genes involved in nitrification (bacterial and archeal *amoA*), nitrogen fixation (*nifH*), and denitrification (*narG, nirS, nirK*, and *nosZ*) as indicators of microbial trait abundances (Wieder et al., 2013), and measured soil variables such as pH, total C and N content, NO_3_^-^, and NH_4_^+^. Soil N isotope composition (δ^15^N) was also analyzed as an indicator of N cycling rates (Högberg, 1997; Hietz et al., 2011; Reverchon et al., 2014). Finally, we discussed the existing relationships between MFG and soil characteristics in tree plantation soils.

## Materials and Methods

### Sampling Design

**FIGURE 1:**
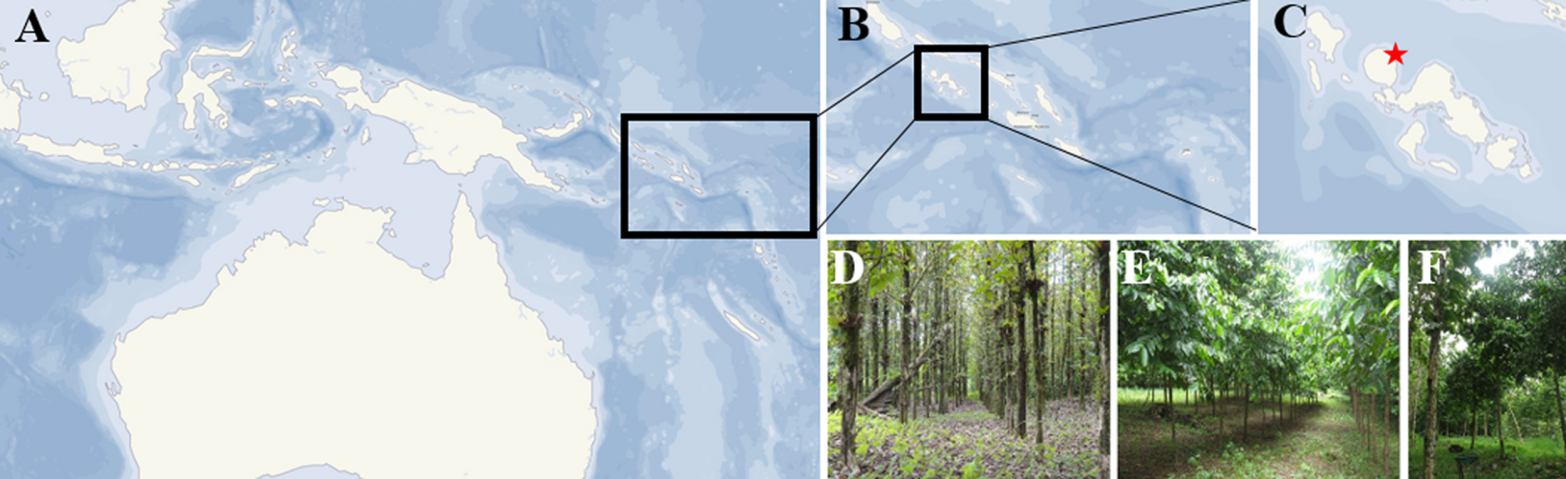
**Smallholder tree plantation systems in Solomon Islands.** The field site is located within the Pacific region **(A)**, in Solomon Islands **(B)**, at Kolombangara, Western Province **(C)**, where teak **(D)**, flueggea **(E)**, and mixed-species **(F)** plantations were established in 2009.

The study site is located at Poitete, on Kolombangara Island, in the Western Province of the Solomon Islands (8° 05′ 16.33″ S and 157° 08′ 46.62″ E; **Figure 1**). The average annual temperature in the area is 28°C and the average rainfall is 3,600 mm, relatively evenly distributed throughout the year (**Supplementary Figure S1**). The soil at the study site is an Oxisol (Vigulu, 2015).

Tree plantations were established in 2009 on land formerly covered with regenerated secondary tropical forests. Plantations consisted of mono-plantations of teak, mono-plantations of flueggea and mixed-species plantations (consecutive rows of teak and flueggea). All plantations were adjacent and therefore established on the same soil type, and had a planting density of 833 stems per hectare (spacing 4 m × 3 m). Three years after planting, teak trees were 16 m high with a diameter at breast height (dbh) of 19 cm, while flueggea trees were 12 m high with a dbh of 13 cm (Vigulu, 2015). The crowns and root systems of both trees were well-developed, with crown radius and root growth of teak trees being larger than those of flueggea (Vigulu, 2015).

Three replicated plots of 24 m × 24 m were established per plantation and a 24-m transect was drawn in the middle of each plot (nine transects in total). In the mixed-species plots, transects were drawn in order to cross alternatively rows of teak and rows of flueggea. Five sampling points were established 4 m apart along each transect. At each sampling point, soil samples were collected on the transect and 4 m perpendicularly on each side of the transect (three samples per sampling point), mixed, and bulked to constitute one composite sample per sampling point. All samples were collected from 0 to 15 cm soil layer, with a shovel. Soil samples were then immediately sieved (2 mm) and refrigerated until analysis (maximum of 5 days). The total number of samples per sampling was 45. Samplings were carried out in December 2012, May 2013, and December 2013.

### Soil Chemical Analyses

Total C, total N, and δ^15^N of soil samples were determined by mass spectrometry (spectrometer GV Isoprime, Manchester, UK), following the procedure described in He et al. (2008). Soil pH was measured in water (1:5 ratio). Soil NH_4_^+^ and NO_3_^-^ concentrations were determined by KCl extraction using a SmartChem 200 Discrete Chemistry Analyser as described in Bai et al. (2012). All soil NO_3_^-^ concentrations were below detection level and were thus not included in the subsequent statistical analyses.

### Quantification of the N-Cycle-Associated MFG

Soil DNA extractions were carried out within 1 week after sampling. DNA was extracted from 0.3 g of each soil sample using the MoBio Powersoil DNA Isolation Kit (MO BIO Laboratories, Carlsbad, CA, USA) according to the manufacturer’s instructions, with a final elution in deionised water. The quantity and quality of the extracted DNA were verified with a Nanodrop spectrophometer (Thermoscientific) and diluted in deionised water (1:10).

The total bacterial community was quantified by quantitative PCR (qPCR) using the V3 hypervariable region of the 16S rRNA gene as a molecular marker. The abundances of functional genes *narG, nirS, nirK, nosZ, nifH*, and bacterial and archeal *amoA* (AOB and AOA respectively) were quantified using the primers and thermal cycling conditions described in Supplementary Table S1. Reactions were carried out in an Eppendorf Mastercycler ep realplex real-time PCR system (Eppendorf, Hamburg, Germany) in duplicate. Quantification was based on the fluorescence intensity of the SYBR Green dye (Takara) during amplification. Standard curves were obtained using 10-fold serial dilutions of plasmid DNA containing cloned *narG, nirS, nirK, nosZ, nifH, amoA* and 16S rRNA genes and spanning seven orders of magnitude. The 20 μL PCR mixture contained 10 μL of SYBR green PCR Master Mix [Takara SYBR Premix Ex Taq (Perfect Real Time)], 0.4 μL of each primer (10 μM) and approximately 8 ng DNA. Melting curves and agarose gels of PCR products were used at the end of each qPCR to check amplification specificity and purity of negative controls. Negative controls gave null or negligible values, and PCR efficiency for the different assays ranged from 90 to 99%. The presence of PCR inhibitors in DNA extracted from soil was estimated by a 1:10 soil DNA dilution; no inhibition was detected. All qPCR reactions were carried out immediately after DNA extraction.

The measured cycle threshold (Ct) values of standards quantification were calibrated by placing the threshold lines at the same level for each gene, to account for the different times at which samples from December 2012, May 2013, and December 2013 were processed for qPCR. Gene data were expressed in number of gene copies ng^-1^ DNA rather than per gram of soil to minimize any bias related to soil DNA extraction efficiency (Čuhel et al., 2010; Correa-Galeote et al., 2013; Rachid et al., 2013).

### Statistical Analyses

A repeated measures two-way analysis of variance (ANOVA) followed by Tukey HSD tests were conducted to detect the effects of plantation type and of sampling time on the measured soil variables and on the abundance of MFG. All data were tested for normality using Shapiro-Wilk normality test and for homogeneity of variance with Levene’s test. Gene abundance data and all soil data except inorganic N were then log-transformed to meet these assumptions. Pearson correlations were performed to analyse the relationships between gene abundances and soil chemical characteristics. A principal component analysis (PCA) was implemented to visualize how MFG abundances were distributed based on plantation type and sampling time. All statistical tests were considered significant at *P* < 0.05. SPSS version 22 was used for all statistical analyses except for PCA which was carried out in R (FactoMine and FactoExtra packages).

## Results

Soil chemical characteristics were significantly influenced by plantation type and sampling time, but the interaction of both factors was not significant, except for soil δ^15^N (**Table 1**). Soil pH was higher under teak plantations than under flueggea or mixed plantations, independent of sampling time (**Table 2**). Conversely, soil total C and N were lower under teak than under mixed plantations, except for total N in samples collected in December 2012. The lowest C:N ratios were also found under teak mono-plantations. The lowest δ^15^N values were found in soil under flueggea mono-plantations, regardless of the time of sampling. The highest δ^15^N values were found for teak plantation soil, although they did not significantly differ from mixed plantations. The effect of sampling time on soil pH, total C and total N was significant, and the highest values were generally found in the last sampling event (December 2013). No differences were found between treatments for soil NH_4_^+^ (**Table 2**).

**Table 1:** *P*-values obtained from a two-way analysis of variance (ANOVA) to detect the effects of plantation type, sampling time, and their interaction on the measured soil variables at Kolombangara, Solomon Islands.

	**df**	**pH**	**Total C**	**Total N**	**C:N**	**NH_4_^+^-N**	**δ^15^N**
	
Plantation type	2	*P* = 0.000	*P* = 0.000	*P* = 0.000	*P* = 0.000	*P* = 0.053	*P* = 0.000
Sampling time	2	*P* = 0.030	*P* = 0.000	*P* = 0.001	*P* = 0.222	*P* = 0.068	*P* = 0.351
Plantation type × sampling time	4	*P* = 0.370	*P* = 0.141	*P* = 0.112	*P* = 0.681	*P* = 0.978	*P* = 0.006

**Table 2:** Soil chemical characteristics measured in different plantation types and at different sampling times at Kolombangara, Solomon Islands.

		**pH (1:5 H_2_O)**	**Total C (g kg^-1^)**	**Total N (g kg^-1^)**
		
December 2012	Teak	6.15 (0.06) a B	60.11 (3.25) b	5.78 (0.33) b
	Flueggea	5.71 (0.14) b AB	75.38 (2.83) a A	6.84 (0.19) a A
	Mixed	5.72 (0.11) b	73.05 (3.33) a AB	6.46 (0.18) ab AB
				
May 2013	Teak	6.24 (0.04) a AB	52.02 (1.60) b	5.10 (0.18) b
	Flueggea	5.47 (0.13) b B	59.46 (3.45) ab B	5.36 (0.27) ab B
	Mixed	5.75 (0.13) b	66.60 (3.27) a B	6.01 (0.28) a B
December 2013	Teak	6.37 (0.06) a A	62.54 (3.40) b	6.03 (0.30) b
	Flueggea	5.96 (0.14) b A	69.96 (3.40) b A	6.31 (0.30) ab A
	Mixed	5.80 (0.10) b	86.00 (4.81) a A	7.47 (0.40) a A
		
		**C:N**	**NH_4_^+^-N (mg kg^-1^)**	**δ^15^N (‰)**
		
December 2012	Teak	10.35 (0.14) b	238.22 (25.54)	6.68 (0.15) a
	Flueggea	11.00 (0.23) ab	258.21 (15.32)	4.04 (0.56) b
	Mixed	11.26 (0.29) a	247.87 (11.75)	5.46 (0.16) a
May 2013	Teak	10.23 (0.10) b	203.56 (15.19)	6.96 (0.09) a
	Flueggea	11.08 (0.27) a	219.08 (18.47)	4.60 (0.55) b
	Mixed	11.09 (0.20) a	228.95 (16.19)	5.28 (0.28) ab
December 2013	Teak	10.37 (0.14) b	202.56 (16.82)	6.47 (0.11) a
	Flueggea	11.12 (0.22) a	224.72 (12.05)	4.45 (0.51) b
	Mixed	11.51 (0.14) a	219.26 (13.92)	4.86 (0.18) ab

*Values between brackets represent standard errors. Small letters indicate significant differences between plantation types for a same sampling time. Capital letters indicate significant differences between sampling times for a same plantation type. No small or capital letters indicate no significant differences between plantation types or sampling times respectively. All differences were considered significant at P < 0.05.*

**FIGURE 2:**
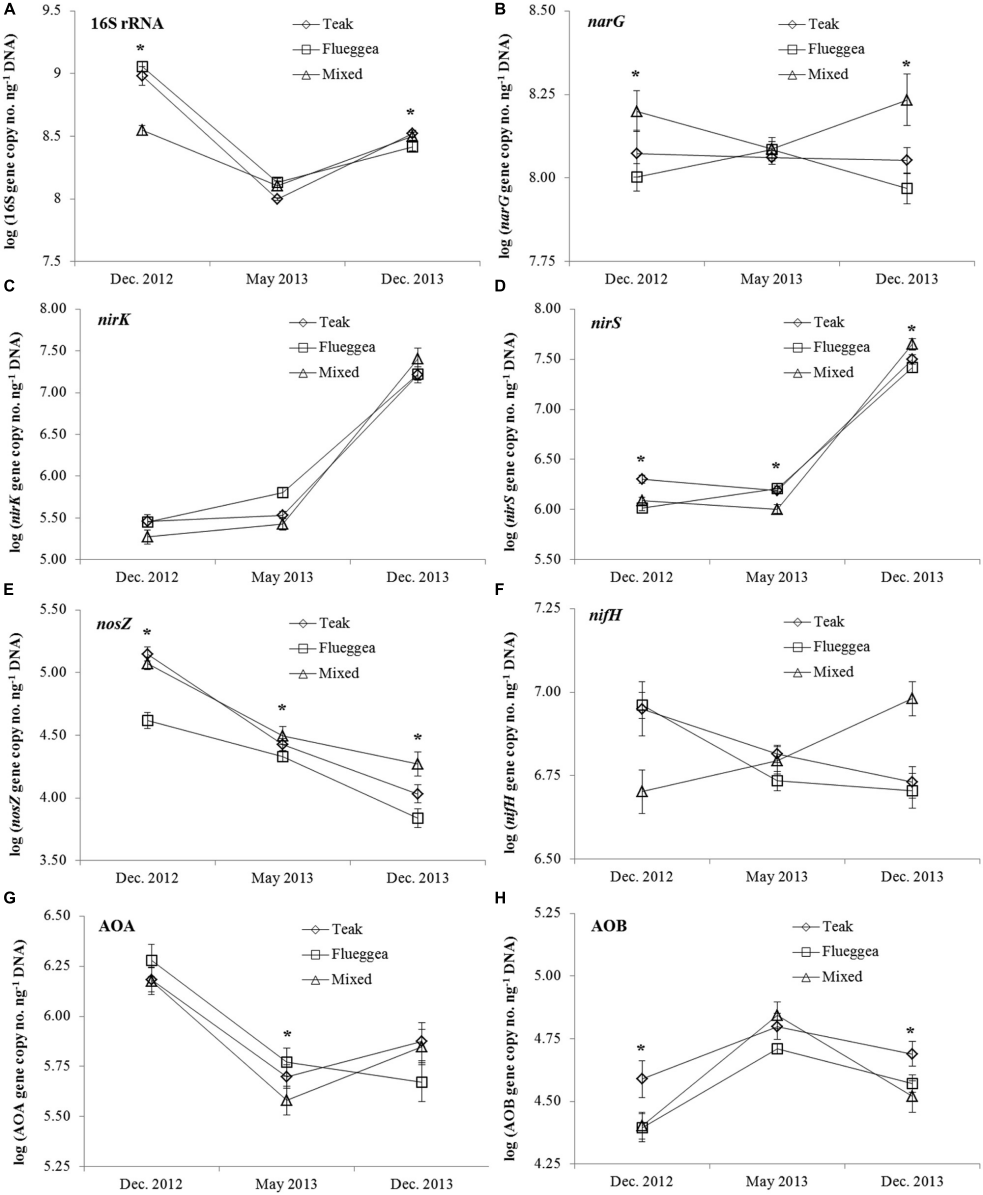
**Abundance of the total bacterial community **(A)** and of the *narG***(B)**, *nirK***(C)**, *nirS***(D)**, *nosZ***(E)**, *nifH***(F)**, AOA **(G)**, and AOB **(H)** genes in soil under plantations of teak (diamonds), flueggea (squares), and mixed species (triangles) at three sampling dates.** Gene abundances are expressed in log (gene copy numbers ng^-1^ DNA). Asterisks indicate significant differences between plantation types (Tukey HSD test, *P* < 0.05).

The abundance of MFG involved in N cycling was significantly influenced by plantation type and sampling time, and the interaction of both factors was generally significant (**Table 3**). The abundance of total bacteria ranged from 1.27 × 10^8^ to 1.27 × 10^9^ copies ng^-1^ DNA and was significantly affected by plantation type in the first and last samplings (**Figure 2A**). The denitrification genes *narG* and *nosZ* were generally most abundant in soils of mixed plantations (**Figures 2B,E**). The abundance of *nirS* was the highest under teak mono-plantations in the first sampling, and under teak and flueggea mono-plantations in the second sampling. However, in the last sampling, *nirS* abundance was higher under mixed plantations than under mono-plantations (**Figure 2D**). The abundance of AOB was higher under teak plantations than under flueggea and mixed plantations in the first and last samplings (**Figure 2H**). No differences were found between plantation types in *nirK, nifH* and AOA, except in the second sampling when AOA abundance was the lowest under mixed plantations (**Figures 2C,F,G**).

**Table 3:** *P*-values obtained from a two-way analysis of variance (ANOVA) to detect the effects of plantation type, sampling time, and their interaction on the abundances of the functional genes involved in soil N cycling at Kolombangara, Solomon Islands.

	**df**	***16S***	***narG***	***nirK***	***nirS***	***nosZ***	***nifH***	**AOA**	**AOB**
	
Plantation type	2	*P* = 0.001	*P* = 0.007	*P* = 0.338	*P* = 0.000	*P* = 0.000	*P* = 0.650	*P* = 0.000	*P* = 0.031
Sampling time	2	*P* = 0.000	*P* = 0.934	*P* = 0.000	*P* = 0.000	*P* = 0.000	*P* = 0.262	*P* = 0.552	*P* = 0.000
Plantation type × sampling time	4	*P* = 0.000	*P* = 0.232	*P* = 0.059	*P* = 0.000	*P* = 0.004	*P* = 0.021	*P* = 0.254	*P* = 0.014

**FIGURE 3:**
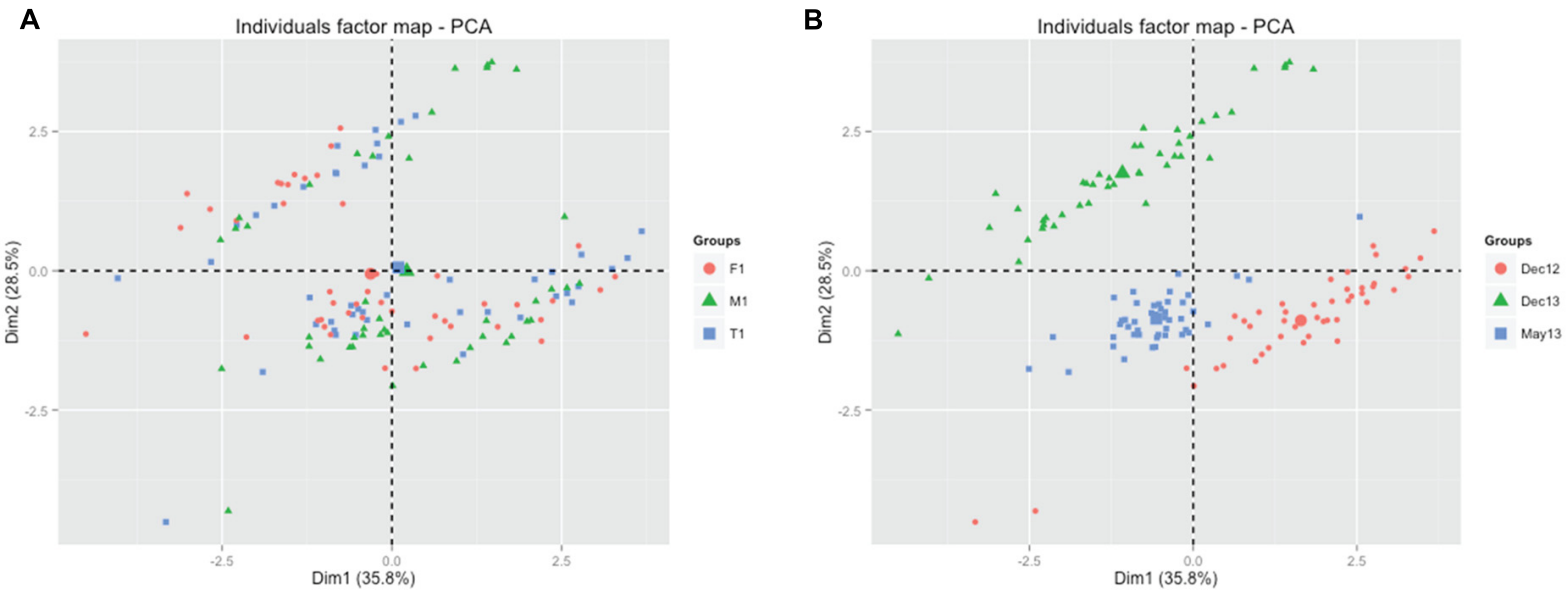
**Principal component analysis (PCA) biplot showing the clustering of microbial functional gene abundances per plantation type **(A)** and per sampling date **(B)**.** T: teak; F: flueggea; M: mixed; Dec12: December 2012; May13: May 2013; Dec13: December 2013.

Sampling time effects on the abundance of MFG were also detected (**Table 3**). The PCA biplot showed that sampling time was the main explanatory variable for gene abundance data, rather than plantation type (**Figures 3A,B**). Generally, the abundances of *nirS* and *nirK* genes were higher in the last sampling event (December 2013) than in the first two samplings (**Figures 2C,D**). On the contrary, the abundances of genes 16S, *nosZ* and AOA were higher in the first sampling (December 2012) than in the two following sampling events (**Figures 2A,E,G**), although ANOVA results showed that this difference was not significant for AOA (Supplementary Table S2). The highest abundances for AOB were found in May 2013 (**Figure 2H**).

**FIGURE 4:**
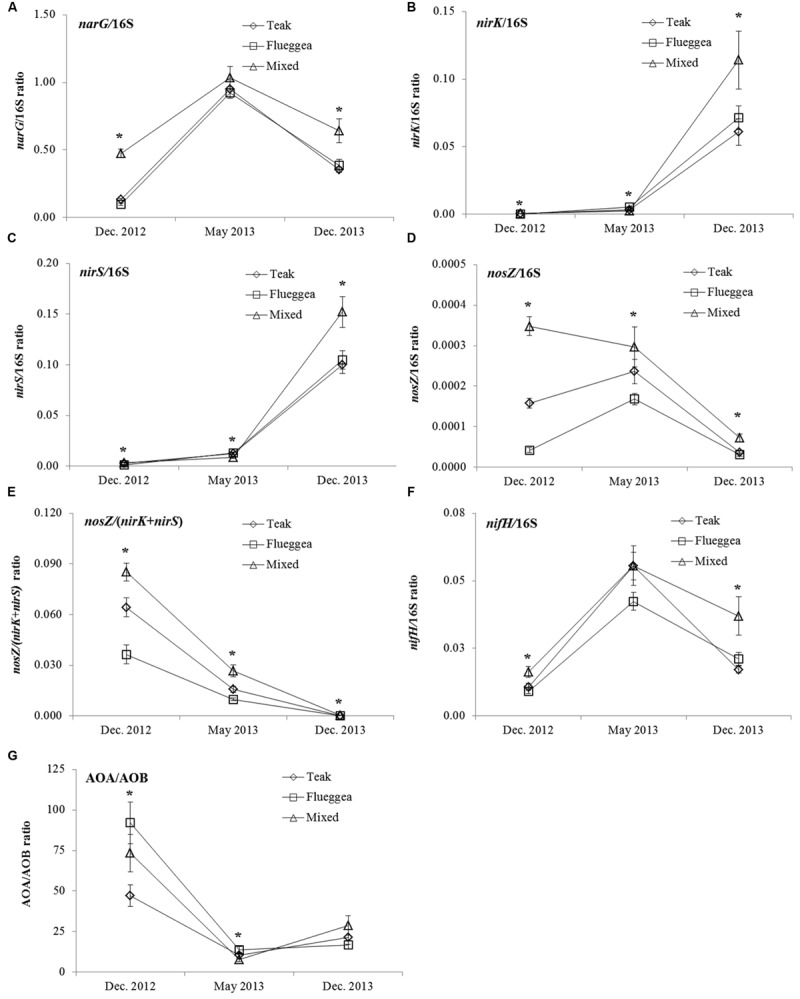
**Relative abundance of the *narG***(A)**, *nirK***(B)**, *nirS***(C)**, *nosZ***(D)** genes, nosZ/(nirK+nirS) ratio **(E)**, relative abundance of the *nifH* gene **(F)**, and AOA/AOB ratio **(G)** in soil under plantations of teak (diamonds), flueggea (squares), and mixed species (triangles) at three sampling dates.** Asterisks indicate significant differences between plantation types (Tukey HSD test, *P* < 0.05).

The relative abundances of *narG, nirK, nirS*, and *nosZ* within the total 16S rRNA gene showed that denitrification genes were generally more abundant under mixed plantations, independently from the sampling time (**Figures 4A–D**). The *nosZ*/(*nirK*+*nirS*) ratio was also larger under mixed plantations than under mono-plantations (**Figure 4E**). The relative abundances of the *nirK* and *nirS* genes revealed a sharp increase in the last sampling while the relative abundance of the *nosZ* gene significantly decreased in December 2013. As a result, the *nosZ*/(*nirK*+*nirS*) ratio was the lowest in the last sampling (**Figure 4E**). When expressed in relation to the size of the entire bacterial community, the *nifH* gene abundance varied depending on plantation type, being larger under mixed plantations than under mono-plantations in the first and last samplings (**Figure 4F**). The AOA/AOB ratio was also influenced by the sampling date, being the largest in the first sampling. In the first two samplings, the AOA/AOB ratio was larger under flueggea than under teak and mixed plantations. However, no significant differences were detected in the AOA/AOB ratio between plantation types in December 2013 (**Figure 4G**).

Pearson correlations showed that soil pH was positively related to the abundance of denitrification genes *nirK* and *nirS*, and to that of AOA (**Table 4**). Soil total C positively correlated with the abundance of total bacteria (16S rRNA), *nirK* and *nirS*, but negatively correlated with the abundance of AOA. Total N positively correlated with the abundance of most N-cycling genes except for *nosZ* and the nitrification genes (AOA and AOB). The C:N ratio strongly and positively correlated with the abundance of *nirK* and *nirS* and negatively correlated with the abundance of 16S rRNA and *nosZ*. Soil δ^15^N only correlated with the abundance of AOA while NH_4_^+^ correlated positively with that of *narG* and nitrification genes AOA and AOB.

**Table 4:** Pearson coefficients for correlation between functional gene abundances and soil characteristics, Kolombangara, Solomon Islands.

	***16S***	***narG***	***nirK***	***nirS***	***nosZ***	***nifH***	***AOA***	***AOB***
	
pH	0.045	0.042	0.204*	0.217*	-0.066	-0.077	0.410**	0.044
C	0.223**	0.159	0.209*	0.207*	0.001	0.168	-0.206*	-0.133
N	0.277**	0.181*	0.219*	0.209*	0.025	0.190*	-0.149	-0.12
C:N	-0.332*	-0.096	0.307**	0.308**	-0.329**	-0.087	-0.139	0.105
δ^15^N	-0.066	0.107	-0.027	0.002	0.129	-0.037	0.228**	0.102
NH_4_^+^	-0.028	0.170*	0.116	0.088	0.016	0.143	0.299**	0.259**

*^∗^Represents significance at *P* < 0.05; ^∗∗^represents significance at *P* < 0.01.*

## Discussion

The objective of this study was to determine how the establishment of mixed plantations would influence soil N pools and transformations, measured through soil chemical properties and MFG abundance. Our results show that soil properties and MFG abundances were both influenced by plantation type. Inter-planting teak with flueggea decreased soil pH and may therefore reduce soil bacterial activities and nutrient cycling rates (Rousk et al., 2009), which could in turn lessen N losses from the system (Xu et al., 2013). Accelerations of N transformation rates and increases in N losses through leaching or denitrification have been associated with enriched soil ^15^N signals (Bai et al., 2015; Reverchon et al., 2015), due to the discrimination against the heavier N isotope during microbially mediated N transformations (Högberg, 1997). Soil δ^15^N values were significantly higher under teak than under flueggea plantation soils and intercropping teak with flueggea seemed to decrease soil δ^15^N, although not significantly, which indicates that N losses through volatilization, leaching, or denitrification could be reduced in mixed plantations.

Soil total C and N content increased when teak was inter-planted with flueggea, which translated into higher C:N ratios in soils from mixed plantations than those in teak mono-plantations. Soil C:N ratio is considered a good indicator of soil fertility as it reflects the coupling between soil organic C (SOC) and total N (Lou et al., 2012; Corral-Fernández et al., 2013). Higher C:N ratios may indicate a slowdown of SOC decomposition and N mineralisation as well as an enhancement of N immobilization (Davidson et al., 2003; Puget and Lal, 2005). Mixed plantations could therefore constitute a way to increase soil C and N stocks without losing the source of income that teak trees represent for local communities. Recently, Lang et al. (2014) showed that N acquisition and retention by subtropical trees were enhanced in mixed plantations when compared with those in mono-plantations, and that this increase could be observed as early as the sapling stage. Whether these positive effects will remain once the trees have reached their full height remains to be investigated, although evidences suggest that complementarity effects among co-occurring species, both at the above- and below-ground level, may promote resource-use efficiency in mixed species stands (Cardinale et al., 2007; Richards et al., 2010). Moreover, aging plantations have been shown to have a positive effect on SOC stocks due to an increase in tree productivity and C belowground allocation with time (Eclesia et al., 2012).

The abundance of MFG associated with N cycling was influenced by plantation type, although the sampling time effect seemed to be stronger than that of tree cover. This is consistent with findings by Yamamura et al. (2013) who reported that tree species scarcely influence N cycling genes and bacterial community structure in tropical plantations. The seasonal differences observed in the present study were unlikely due to variations in rainfall or temperature, since climatic conditions in Solomon Islands are relatively homogeneous through the year. However, seasonal variations in soil conditions, affecting plant growth and productivity, bacterial community composition, and ultimately ecosystem processes, could have altered microbial traits. Seasonal differences were detected in pH, total C and total N. Additionally, Rasche et al. (2011) showed that seasonal dynamics of microbial functional groups involved in N cycling were strongly related with seasonal changes in soil labile C and N, and in general with resource availability. This seasonality was especially apparent in the relative abundances of denitrification genes, with the relative abundance of nitrite reductase genes (*nirK* and *nirS*) showing a drastic increase in the last sampling. As a consequence, the *nosZ*/(*nirK*+*nirS*) ratio presented a sharp decrease at the same period, as did the AOA/AOB ratio. Denitrifier and nitrifier microorganisms (bacteria and archaea) have been shown to be particularly affected by seasonal shifts in the soil environment, such as soil temperature or moisture (Shen et al., 2008; Stres et al., 2008), which are closely related with resource availability and labile C pools (Bell et al., 2009). Seasonal patterns of tree belowground C allocation may also contribute to temporal variations in microbial dynamics (Kaiser et al., 2011; Churchland and Grayston, 2014). More recently, Graham et al. (2014) reported that temporal dynamics were critical to unraveling the relationships between soil properties and gene abundance data, and thus recommended to examine seasonal samples separately in order to develop accurate models of ecosystem functioning.

Generally, the abundance of MFG associated with denitrification (*narG, nosZ*, and *nirS*) was larger in soils from mixed plantations than in those from teak mono-plantations, especially in the last sampling. Gene copy numbers in MFG only vary from 1 to 3 in bacterial cells, while the number of 16S rRNA gene copies range from 1 to 13 per cell (Fogel et al., 1999). Therefore, whilst the observed differences in 16S rRNA gene abundance between tree plantation systems could have been due to differences in species composition of the bacterial community (Liu et al., 2015), our results indicate that denitrifier communities were more abundant under mixed plantations, which is confirmed by the relative abundances of denitrification genes. An increase in the abundance of *narG* (nitrate reductase) and *nosZ* (nitrous oxide reductase) may reduce the negative environmental impacts associated with leaching of NO_3_^-^ and with N_2_O emissions (Henry et al., 2006). The larger *nosZ*/(*nirK*+*nirS*) ratio under mixed plantations indicated an enrichment in *nosZ*-harboring denitrifiers when teak was inter-planted with flueggea, which could in turn have implications for N_2_O/N_2_ emissions from tree plantations (Liu et al., 2015). The measurement of atypical *nosZ* abundance, which was not quantified in this study, would also complement the analysis of bacterial and archeal contributions to N_2_O emissions from plantation soils (Sanford et al., 2012).

Overall, the increase in denitrification gene abundances in mixed plantations may indicate larger denitrifying bacterial communities when teak is inter-planted with flueggea (Baudoin et al., 2009). Denitrifier bacteria have been reported to be influenced by the quantity and composition of organic compounds emitted by roots or resulting from the decomposition of organic residues (Kandeler et al., 2006; Henry et al., 2008). Mixed plantations, by promoting the diversity of bioavailable C sources associated with root exudates and litterfall decomposition, could sustain more abundant denitrifier communities than mono-plantations. The abundance of most denitrification genes correlated with soil total N content and C:N ratio, and *nirK*/*nirS* gene abundances positively correlated with total C content. These findings are consistent with results from previous studies (Lindsay et al., 2010; Ducey et al., 2013) and confirm the heterotrophic nature of denitrifier bacterial communities (Baudoin et al., 2009). Furthermore, soil total C, total N, and C:N were all influenced by plantation type, which suggests that tree species mixture is likely to be a significant driver of the denitrifier community through the modification of soil properties.

No differences in *nifH* abundance were detected between plantation types. This is consistent with findings from Rachid et al. (2013) who showed that soil free-living nitrogen-fixing bacteria were not altered by the establishment of mixed plantations. However, in the first and last samplings, the relative abundance of the *nifH* gene was larger in mixed plantations than in mono-plantations, which reflects an enrichment in N-fixing bacteria when teak was inter-planted with flueggea and could, over time, increase soil N content (Lammel et al., 2015). Nitrifier communities were also affected by plantation type, as shown by the significant decrease in AOB abundance under mixed plantations compared with teak mono-plantations. This reduction of AOB abundance in mixed plantations did not translate into alterations in soil NH_4_^+^ concentrations, although both AOA and AOB genes correlated significantly with soil NH_4_^+^. The lack of alteration in soil NH_4_^+^ may be due to the dominance of AOA in the ammonia oxidation process, as indicated by AOA:AOB ratios higher than 10 in almost all treatments (Prosser and Nicol, 2012). Furthermore, the abundance and community structure of nitrifiers have been shown to be highly dependent upon pH (Wessén et al., 2011; Levy-Booth et al., 2014), and different phylotypes of bacterial and archaeal ammonia oxidisers are selected in soils with different pH (Nicol et al., 2008), which in turn could affect nitrification rates.

Differences in N pools and transformations between different tree plantation systems may be attributed to a combination of alterations in the soil physico-chemical environment and in soil microbial communities (Anderson et al., 2014). Tree mycorrhizal symbionts could further influence N cycling by accessing different soil N sources or by enhancing the immobilization of inorganic N (Michelsen et al., 1996). While teak has been reported to form arbuscular mycorrhizal fungal associations (Rajan et al., 2000), the mycorrhizal status of flueggea is yet to be studied and may have implications for species interactions in these mixed plantations. Moreover, bacterial taxonomic and functional diversity could be affected by tree species composition directly through changes in the amount and composition of root exudates and litterfall, and indirectly through changes in soil parameters (da C Jesus et al., 2009; Rousk et al., 2010). The significant correlations found in the present study between some soil factors and MFG abundances confirmed the influence of plantation type on soil bacterial communities through the modification of the soil environment. However, there was no strong coupling between gene abundances and the measured soil parameters. This lack of correspondence may be due to different factors. First, process rates may be more readily influenced by changes in tree cover than taxonomic or functional diversity of bacterial communities (Rocca et al., 2015). Moreover, changes in soil parameters may have a stronger effect on the relative proportion of nitrifier/denitrifier bacteria than on MFG abundance (Anderson et al., 2014). Finally, the presence of functional genes does not always indicate an active bacterial community (Levy-Booth et al., 2014) and expression of MFG measured through mRNA transcripts, rather than MFG abundance, could be a better predictor of microbial functional capacity and hence N transformation rates in soil (Wood et al., 2015).

Due to the lack of proper conditions available at the sampling site, it was not possible to freeze the samples and maintain them at -80°C until DNA extraction. Sample storage at 4°C for up to 5 days prior to analysis may have induced changes in bacterial community structure and MFG abundances, as reported by Ott et al. (2004) for fecal samples. However, recent findings by Lauber et al. (2010) and Rubin et al. (2013) showed that neither storage time nor storage temperature drastically affected microbial community composition and structure from soil samples. In addition, storage conditions were consistent across our three sampling events, thereby reducing any bias resulting from sample collection and preparation.

With teak native forests disappearing, teak plantations are expanding and now cover 4.3⋅10^6^ ha (Fernández-Moya et al., 2014). Establishing mixed plantations as alternatives to mono-species teak stands would guarantee economic returns without a subsequent impoverishment of soil fertility. Our results show that inter-planting teak with flueggea would benefit soil quality and sustainability by increasing soil C and N pools, which we hypothesized could be due to a decrease in potential losses from leaching and N_2_O emissions or to higher inputs from flueggea trees. In addition, soil quality under mixed plantations would be enhanced by the diversity of bioavailable C compounds from root exudates and leaf litter. Kirby and Potvin (2007) reported a positive correlation between biodiversity and organic C in soils, thereby suggesting that mixed plantations could improve soil physical structure (Xing et al., 2004). Our findings are further supported by productivity and aboveground nutrient accumulation data showing the potential for teak to be grown in mixed plantations (Vigulu, 2015). Further research should concentrate on assessing other mixed plantation models under different sets of environmental conditions, and on incorporating microbial dynamics into plantation management to improve productivity while mitigating soil fertility loss.

## Conflict of Interest Statement

The authors declare that the research was conducted in the absence of any commercial or financial relationships that could be construed as a potential conflict of interest.

## Supplementary Material

Figure S1 **Average rainfall data at Kolombangara, Western Province, Solomon Islands, from 1993 to 2014**.
