# Supplementary figures and images for: Tree Plantation Systems Influence Nitrogen Retention and the Abundance of Nitrogen Functional Genes in the Solomon Islands
Source: Front Microbiol. 2015 Dec 22;6:1439. doi: 10.3389/fmicb.2015.01439 (PMC4686685; doi:10.3389/fmicb.2015.01439)

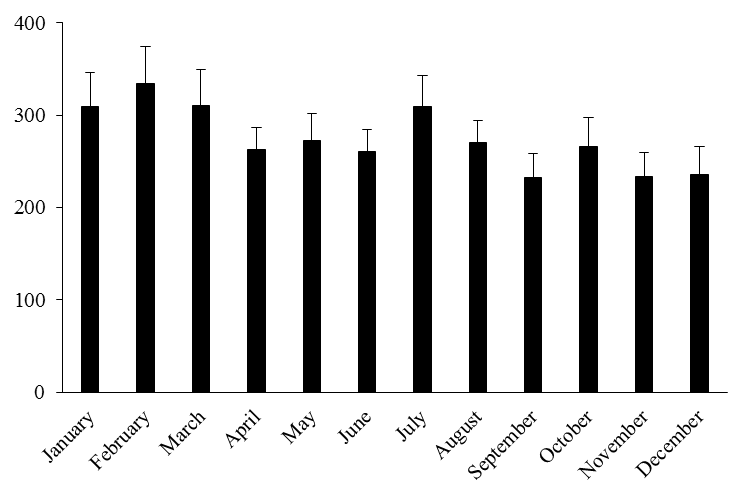

Supplement: Figure S1 — Average rainfall data at Kolombangara, Western Province, Solomon Islands, from 1993 to 2014. [file Image_1.TIF]
