# Supplementary material for: Tree Plantation Systems Influence Nitrogen Retention and the Abundance of Nitrogen Functional Genes in the Solomon Islands
Source: Front Microbiol. 2015 Dec 22;6:1439. doi: 10.3389/fmicb.2015.01439 (PMC4686685; doi:10.3389/fmicb.2015.01439)
Supplement: Supplementary file 2 [file Table_1.DOCX]

**Supplementary Table 1**. Primers and thermal profiles used for real-time PCR quantification of the studied genes.

| Target gene | Primers | References | Denaturation time at 95˚C (s) | Annealing temperature and time | Elongation temperature and time | Number of cycles |
| --- | --- | --- | --- | --- | --- | --- |
| 16S rRNA | 338F / 518R | Liu et al., 2013 | 10 | 55˚C, 15 s | 72˚C, 30 s | 40 |
| *amoA* (AOA) | CrenamoA23F / CrenamoA616R | Liu et al., 2013 | 15 | 55˚C, 30 s | 72˚C, 45 s | 40 |
| *amoA* *(*AOB*)* | AmoA1F / AmoA2R | Rotthauwe et al., 1997 | 15 | 55˚C, 30 s | 72˚C, 45s | 40 |
| *narG^a^* | narGG-R / narGG-F | Bru et al., 2007 | 30 | 58˚C, 30 s | 72˚C, 30s | 35 |
| *nifH^b^* | Pol-F / Pol-R | Severin et al., 2010 | 15 | 54°C, 30 s | 72°C, 30 s | 35 |
| *nirK^a^* | nirK876 / nirK1040 | Henry et al., 2004 | 30 | 58˚C, 30 s | 72˚C, 30 s | 35 |
| *nirS^a^* | nirS4QF / nirS6QR | Kandeler et al., 2006 | 30 | 58˚C, 30 s | 72˚C, 30 s | 35 |
| *nosZ^c^* | nosZ2F / nosZ2R | Henry et al., 2006 | 30 | 60˚C, 30 s | 72˚C, 30 s | 35 |

^a^Touch down starting at 63˚C temperature decrease of 1˚C per cycle for 6 cycles

^b^Touch down starting at 59˚C temperature decrease of 1˚C per cycle for 6 cycles

^c^Touch down starting at 65˚C temperature decrease of 1˚C per cycle for 6 cycles
