# Supplementary material for: Tree Plantation Systems Influence Nitrogen Retention and the Abundance of Nitrogen Functional Genes in the Solomon Islands
Source: Front Microbiol. 2015 Dec 22;6:1439. doi: 10.3389/fmicb.2015.01439 (PMC4686685; doi:10.3389/fmicb.2015.01439)
Supplement: Supplementary file 3 [file Table_2.DOCX]

**Supplementary Table 2.** Functional gene abundances (gene copy numbers ng^-1^ DNA) measured in different plantation types and at different sampling times at Kolombangara, Solomon Islands. Values between brackets represent standard errors. Small letters indicate significant differences between plantation types for a same sampling time. Capital letters indicate significant differences within sampling times for a same plantation type. All differences were considered significant at *P* < 0.05.

|  |  | ***16S*** | | ***narG*** | | ***nirK*** | | ***nirS*** | |
| --- | --- | --- | --- | --- | --- | --- | --- | --- | --- |
| **Dec. 2012** | Teak | 1.12E+09 | (1.05E+08) aA | 1.41E+08 | (1.34E+07) ab | 3.43E+05 | (3.18E+04) B | 2.17E+06 | (1.80E+05) aB |
|  | Flueggea | 1.27E+09 | (1.42E+08) aA | 1.10E+08 | (8.53E+06) b | 3.21E+05 | (3.15E+04) C | 1.07E+06 | (4.60E+04) bC |
|  | Mixed | 3.69E+08 | (1.73E+07) bA | 1.81E+08 | (1.53E+07) a | 2.36E+05 | (2.58E+04) B | 1.29E+06 | (6.90E+04) bB |
|  |  |  |  |  |  |  |  |  |  |
| **May 2013** | Teak | 1.27E+08 | (8.04E+06) C | 1.17E+08 | (4.17E+06) | 4.01E+05 | (5.95E+04) B | 1.58E+06 | (6.45E+04) aC |
|  | Flueggea | 1.42E+08 | (9.39E+06) C | 1.25E+08 | (5.29E+06) | 7.30E+05 | (7.30E+04) B | 1.69E+06 | (1.02E+05) aB |
|  | Mixed | 1.38E+08 | (1.09E+07) B | 1.30E+08 | (1.04E+07) | 3.39E+05 | (4.41E+04) B | 1.10E+06 | (9.19E+04) bB |
|  |  |  |  |  |  |  |  |  |  |
| **Dec. 2013** | Teak | 3.42E+08 | (1.59E+07) aB | 1.20E+08 | (7.25E+06) b | 2.23E+07 | (3.10E+06) A | 3.42E+07 | (2.63E+06) bA |
|  | Flueggea | 2.71E+08 | (1.21E+06) bB | 1.05E+08 | (1.26E+07) b | 1.92E+07 | (1.75E+06) A | 2.84E+07 | (2.09E+06) bA |
|  | Mixed | 3.35E+08 | (2.08E+07) aA | 2.26E+08 | (3.36E+07) a | 4.30E+07 | (7.41E+06) A | 5.05E+07 | (4.85E+06) aA |
|  |  |  |  |  |  |  |  |  |  |
|  |  | ***nosZ*** | | ***nifH*** | | **AOA** | | **AOB** | |
| **Dec. 2012** | Teak | 1.57E+05 | (1.11E+04) aA | 1.18E+07 | (1.80E+06) | 1.74E+06 | (1.60E+05) | 4.62E+04 | (4.42E+03) aB |
|  | Flueggea | 4.98E+04 | (5.71E+03) bA | 1.02E+07 | (9.79E+05) | 2.37E+06 | (2.75E+05) | 2.93E+04 | (3.46E+03) bC |
|  | Mixed | 1.30E+05 | (9.50E+03) aA | 6.01E+06 | (6.11E+05) | 1.74E+06 | (1.54E+05) | 2.79E+04 | (2.00E+03) bB |
|  |  |  |  |  |  |  |  |  |  |
| **May 2013** | Teak | 2.90E+04 | (2.31E+03) abB | 6.80E+06 | (3.59E+05) | 5.92E+05 | (7.92E+04) ab | 1.09E+05 | (5.14E+04) A |
|  | Flueggea | 2.31E+04 | (1.63E+03) bB | 5.65E+06 | (3.07E+05) | 2.37E+06 | (2.75E+05) a | 5.27E+04 | (2.05E+03) A |
|  | Mixed | 4.13E+04 | (8.39E+03) aB | 6.81E+06 | (6.87E+05) | 4.56E+05 | (4.49E+04) b | 7.90E+04 | (7.89E+03) A |
|  |  |  |  |  |  |  |  |  |  |
| **Dec. 2013** | Teak | 1.28E+04 | (1.18E+03) bC | 5.85E+06 | (4.30E+05) | 9.74E+05 | (1.11E+05) | 5.38E+04 | (4.60E+03) aAB |
|  | Flueggea | 8.66E+03 | (1.09E+03) bC | 5.72E+06 | (6.16E+05) | 6.28E+05 | (6.82E+04) | 3.90E+04 | (2.13E+03) bB |
|  | Mixed | 2.55E+04 | (3.30E+03) aC | 1.13E+07 | (1.64E+06) | 9.00E+05 | (1.08E+05) | 4.07E+04 | (6.77E+03) bB |
